# Hyperfructosemia in sleep disordered breathing: metabolome analysis of Nagahama study

**DOI:** 10.1038/s41598-023-40002-1

**Published:** 2023-08-05

**Authors:** Yoshinari Nakatsuka, Kimihiko Murase, Kazuhiro Sonomura, Yasuharu Tabara, Tadao Nagasaki, Satoshi Hamada, Takeshi Matsumoto, Takuma Minami, Osamu Kanai, Hirofumi Takeyama, Hironobu Sunadome, Naomi Takahashi, Isuzu Nakamoto, Kiminobu Tanizawa, Tomohiro Handa, Taka-Aki Sato, Naoko Komenami, Tomoko Wakamura, Satoshi Morita, Osamu Takeuchi, Takeo Nakayama, Toyohiro Hirai, Yoichiro Kamatani, Fumihiko Matsuda, Kazuo Chin

**Affiliations:** 1 Department of Respiratory Care and Sleep Control Medicine, Graduate School of Medicine, Kyoto University, Kyoto, Japan; 2 Center for Genomic Medicine, Graduate School of Medicine, Kyoto University, Kyoto, Japan; 3 Graduate School of Public Health, Shizuoka Graduate University of Public Health, Shizuoka, Japan; 4 Department of Advanced Medicine for Respiratory Failure, Graduate School of Medicine, Kyoto University, Kyoto, Japan; 5 Department of Respiratory Medicine, Saiseikai Noe Hospital, Osaka, Japan; 6 Department of Primary Care and Emergency Medicine, Graduate School of Medicine, Kyoto University, Kyoto, Japan; 7 Division of Respiratory Medicine, Center for Respiratory Diseases, National Hospital Organization Kyoto Medical Center, Kyoto, Japan; 8 Nursing Science, Human Health Sciences, Graduate School of Medicine, Kyoto University, Kyoto, Japan; 9 Department of Respiratory Medicine, Graduate School of Medicine, Kyoto University, Kyoto, Japan; 10 Life Science Research Center, Technology Research Laboratory, Shimadzu Corporation, Kyoto, Japan; 11 Department of Food and Nutrition, Kyoto Women’s University, Kyoto, Japan; 12 Department of Biomedical Statistics and Bioinformatics, Graduate School of Medicine, Kyoto University, Kyoto, Japan; 13 Department of Medical Chemistry, Graduate School of Medicine, Kyoto University, Kyoto, Japan; 14 Department of Health Informatics, Kyoto University School of Public Health, Kyoto, Japan; 15 Department of Sleep Medicine and Respiratory Care, Division of Respiratory Medicine, Nihon University of Medicine, 1-30, Uemachi Otaniguchi Itabashi-Ku, Tokyo, 173-8610 Japan

**Keywords:** Respiratory tract diseases, Hypoxia, Epidemiology

## Abstract

Sleep disordered breathing (SDB), mainly obstructive sleep apnea (OSA), constitutes a major health problem due to the large number of patients. Intermittent hypoxia caused by SDB induces alterations in metabolic function. Nevertheless, metabolites characteristic for SDB are largely unknown. In this study, we performed gas chromatography-mass spectrometry-based targeted metabolome analysis using data from The Nagahama Study (n = 6373). SDB-related metabolites were defined based on their variable importance score in orthogonal partial least squares discriminant analysis and fold changes in normalized peak-intensity levels between moderate-severe SDB patients and participants without SDB. We identified 20 metabolites as SDB-related, and interestingly, these metabolites were frequently included in pathways related to fructose. Multivariate analysis revealed that moderate-severe SDB was a significant factor for increased plasma fructose levels (β = 0.210, *P* = 0.006, generalized linear model) even after the adjustment of confounding factors. We further investigated changes in plasma fructose levels after continuous positive airway pressure (CPAP) treatment using samples from patients with OSA (n = 60) diagnosed by polysomnography at Kyoto University Hospital, and found that patients with marked hypoxemia exhibited prominent hyperfructosemia and their plasma fructose levels lowered after CPAP treatment. These data suggest that hyperfructosemia is the abnormality characteristic to SDB, which can be reduced by CPAP treatment.

## Introduction

Sleep disordered breathing (SDB) and its most frequent subtype obstructive sleep apnea (OSA) are prevalent, and moderate to severe SDB is found in 20 to 40% of adults worldwide^1–3^. Recent data showed that 936 and 425 million adults aged 30–69 have mild and moderate to severe OSA, respectively^4^. While OSA causes various symptoms such as daytime sleepiness, it is also frequently associated with multiple complications including cardiovascular diseases (CVD) such as stroke and myocardial infarction^5–8^, which culminates in a significant increase in mortality for patients with SDB compared to healthy people^9^.

Pathophysiologically, OSA induces intermittent hypoxia, and the systemic response to intermittent hypoxia was suggested to constitute the basis for complications of OSA^10^. Recent studies highlighted the disruption of metabolic systems as one of the hallmark abnormalities downstream of intermittent hypoxia^10,11^. As the abnormalities in the metabolism are strongly associated with the development of CVD, it is of great clinical significance to identify the metabolic pathways associated with OSA^7^. Nevertheless, the metabolic pathways that are characteristically associated with SDB remain unclear.

Recent advances in mass-spectroscopic analyses enable the acquisition of the comprehensive data on plasma metabolite levels by metabolome analysis, which serves as a strong tool for the identification of key pathophysiological mechanisms of diseases^10,12^. Metabolome analyses revealed that tissue hypoxia could enhance glycolytic metabolism, which might result in alteration in the levels of glycolytic intermediates^12,13^. This knowledge suggests that these metabolites are potential targets for investigation, and to date, a number of studies have attempted to reveal metabolic signatures in SDB patients by metabolomic analysis^14–17^. However, a large obstacle in the metabolic characterization of SDB patients is the confounding factors possessed by the majority of SDB patients. SDB patients are frequently obese, preferentially male, and tend to be elderly. These factors can strongly affect metabolic characteristics and interfere with the evaluation of specific associations between SDB and metabolic pathways. One strategy to control for these confounding factors is to perform a study that includes large number of participants with diverse background and adjust for those factors. However, studies conducted so far included a relatively small number of patients, and it has been difficult to adequately control for confounding factors.

In this study we hypothesized that SDB is associated with specific metabolic pathways downstream of intermittent hypoxia and independent from other confounding factors. By using targeted plasma metabolome data measured by gas-chromatography mass-spectrometry (GC–MS) in a large-scale community-based cohort, we compared individuals with and without SDB for differences in metabolite levels. In addition, we investigated changes in the identified metabolite after continuous positive airway pressure (CPAP) treatment using our institutional cohort data.

## Methods

### Study design and participants

We performed a retrospective cross-sectional study using the information obtained from the second visit dataset of the Nagahama Prospective Genome Cohort for the Comprehensive Human Biology (The Nagahama Study)^18,19^. This community-based genome cohort study recruits participants from the general population of Nagahama, a rural Japanese city with 125 000 residents, between 2008 and 2010. Residents who did not have physical impairment or dysfunction and between 30 and 74 years of age were considered eligible for recruitment. A total of 9804 participants were recruited. Among them, the following participants were excluded; 14 withdrew consent and 26 having a different ethnic background according to genetic analysis. The remaining 9764 participants were treated as the baseline population, and invited for the second health survey 5 years after the baseline evaluation. Among them, the second health survey was performed on 8289 people. In addition, 1561 participants were newly recruited during second survey period, and the second-visit dataset of the Nagahama study consisted of 9850 participants^18^. Participants were included in the present study when data from at least 2 nights were available. Participants were excluded if plasma samples were not available for analysis or if they were already receiving treatment for OSA (e.g., continuous positive airway pressure (CPAP) treatment or oral appliance). Participants were instructed to skip the meal before the visit.

We also analyzed an institutional retrospective longitudinal cohort of clinically stable patients older than 20 years diagnosed with OSA by polysomnography at the Sleep Laboratory of Kyoto University Hospital between 2012 and 2014. The patients in this institutional cohort underwent polysomnography at diagnosis and a follow-up study three months after CPAP treatment to assess the treatment efficacy. Patients lacking samples at the time of the diagnosis or the follow-up study were excluded, as were those with acute infection or active malignancies. The meals during hospital stay were decided prior to admission based on their age, sex and complications such as diabetes. They did not have choices of contents that might change the amount of nutrients (e.g. calories, lipids or carbohydrates). Patients finished the evening meal before 7 pm and then undergo polysomnography, during which they were not allowed to eat. In both cohorts, written informed consent was obtained from all participants. The Ethics Committee of Kyoto University Graduate School of Medicine approved these two cohort studies (G0278 and E1475). These studies were performed in accordance with the Declaration of Helsinki.

### Sleep data acquisition and analysis

In the Nagahama study, SDB was diagnosed based on the actigraph-adjusted oxygen desaturation index of 3% (Acti-ODI3%) calculated from the continuous SpO_2_ data at night, derived from pulse oximetry (PULSOX-Me300; Konica Minolta, Inc., Tokyo, Japan) and adjusted using the objective sleep duration obtained using an Actiwatch 2 or the Actiwatch Spectrum Plus wrist actigraph (Philips Respironics, Murrysville, PA, USA)^3^. In our previous data, Acti-ODI3% was more comparable to the apnea–hypopnea index (AHI) derived from attended polysomnography in 32 patients (r = 0.99, *P* < 0.001; AHI = Acti-ODI3%*1.04 + 1.45) than simply-measured ODI3% without actigraphy-modification (r = 0.92, *P* < 0.001; AHI = usual ODI3%*1.27 + 2.06)^19^. Participants were divided into three groups based on their Acti-ODI3% values according to the American Association of Sleep Medicine guidelines: normal group: < 5/h, mild group: 5 to < 15/h and moderate-severe group, ≥ 15/h. OSA in the institutional cohort was diagnosed according to the American Association of Sleep Medicine guidelines based on polysomnography results.

### Sample and data collection

Plasma samples in the Nagahama study were collected at study visits and stored at − 80 °C until analysis. Information including the participants’ sex, menopausal status, past medical histories, comorbid diseases, currently prescribed medications, smoking history, and the timing of the last meal were collected. Body weight, height, blood pressure, and pulse rate were evaluated at the study visit. We measured the maximum value of intima-media thickness of the common carotid artery (CCA-IMT-max) by ultrasonography through the lateral approach.

In the institutional cohort, plasma samples were collected at the time of diagnosis and at each follow-up study. Plasma samples were collected after the completion of polysomnography and before breakfast.

In total of 121 plasma metabolites were measured by GCMS-QP2010 Ultra (Shimadzu Corp., Kyoto, Japan)^20^. To analyze the institutional cohort, we evaluated the concentration of fructose in each sample using a single-point calibration curve derived from concentration-determined D-Fructose 13C6 (ISOTEC, Miamisburg, OH, USA).

### Diagnosis of complications

Hypertension was considered when participants were taking blood pressure-lowering medicine or had an on-site measured systolic blood pressure ≥ 140 mmHg and/or diastolic blood pressure ≥ 90 mmHg. Diabetes mellitus was indicated by hemoglobin A1c ≥ 6.5% or treatment with oral antihyperglycemic drugs and/or insulin. Dyslipidemia was considered present according to plasma LDL-cholesterol levels > 140 mg/dl, plasma HDL-cholesterol levels < 40 mg/dl, plasma triglyceride levels > 150 mg/dl or the use of anti-dyslipidemia drugs. Female participants < 60 years old were considered as pre-menopausal if they replied “no” to the question about menopausal status. The diagnosis of metabolic syndrome was made according to Japanese criteria^21^. Briefly, criteria were waist circumference ≥ 85 cm (male) or ≥ 90 cm (female) accompanied by two or more of the following factors: 1, systolic blood pressure ≥ 130 mmHg and/or diastolic blood pressure ≥ 85 mmHg; 2, plasma triglycerides ≥ 150 mg/dl and/or plasma HDL levels < 40 mg/dl, and 3, fasting blood glucose ≥ 100 mg/dl.

### GC–MS

Sample preparation for GC–MS was performed as described previously ^20^. In short, 50 μl of plasma were mixed with the extraction solvent containing the internal standard (2-isopropylmalic acid). After centrifugation, the supernatant was collected and dried with a centrifugal evaporator. After two derivatization procedures (oximation and trimethylsilyl derivatization), the extract was injected into the GC–MS system. GC–MS measurement using GCMS-QP2010 ultra (Shimadzu Corp., Kyoto, Japan) was performed according to a previous study^20^. Chromatogram acquisition, detection of mass spectral peaks, and their waveform processing were performed using Shimadzu GC/MS solution software Version 2.71 (Shimadzu Corp., Kyoto, Japan). Small polar metabolites were identified using a commercially available GC–MS Metabolite Mass Spectral Database (Shimadzu Corp., Kyoto, Japan). Peak area of each metabolite was calculated, and then normalized using the internal standard peak. In the analysis of the institutional cohort, we evaluated the actual concentration of each sample by using a single-point calibration curve derived from concentration-determined D-Fructose 13C6 (ISOTEC, Miamisburg, OH, USA).

### Statistical analyses

First, we sought to identify metabolites characteristic of SDB patients using the metabolome analysis platform Metaboanalyst 4.0^22^. Because the effect of SDB on metabolic complications was strongly noted in those with moderate to severe SDB^23^, we compared normal group with moderate-severe group. To determine metabolites characteristic of SDB, we employed two strategies. The first was orthogonal partial least squares discriminant analysis (OPLS-DA), a multivariate analysis which builds a model to differentiate participants without SDB from participants with moderate-severe SDB based on normalized peak-intensity of plasma metabolites^24,25^. Normalization of peak-intensity was performed by log-transformation and the auto-scaling method. The Variable Importance for Projection (VIP) score indicates the degree of contribution for each metabolite to the model of OPLS-DA. According to a previous study^26^, a VIP score ≥ 0.9 was considered as metabolites differentiating SDB patients from normal participants. The second strategy was to screen metabolites exhibiting large differences in peak-intensity between the normal group and moderate-severe SDB group. We extracted the top 30 metabolites ranked according to fold changes in normalized peak intensity among the metabolites with a false discovery rate adjusted *P*-value < 0.05 by using unpaired t-test. We then identified metabolites that were consistently selected by these two strategies and considered them as SDB-related metabolites. Then we put them into pathway analysis. Over-representation analysis with Fisher’s exact test was used to analyze if the number of SDB-related metabolites within the individual pathway is significantly larger than expected^27^. We also performed pathway topology analysis based on out-degree centrality, which estimates the impact of the metabolite within each pathway^27^. KEGG database was used as the reference^27^.

After identification of fructose as a candidate metabolite that is affected by SDB, we examined its clinical relevance using EZR (Saitama Medical Centre, Jichi Medical University, Saitama, Japan), which is a graphical user interface for R (The R Foundation for Statistical Computing, Vienna, Austria)^28^. Raw peak intensity levels of plasma fructose, hypoxanthine, and uric acid were compared between groups by the Kruskal Wallis test, and Holm’s post-hoc test was used to compare two out of three groups. To adjust the effects of confounding factors for the elevation of plasma peak intensity levels of these metabolites, we used a generalized linear model by setting the link function as family = Gamma(log). Two models were employed for the analyses. Model 1 included the following factors as explanatory variables: age, BMI, sex (female), current smoker status, diabetes, hypertension, dyslipidemia and waist circumference. Model 2 included metabolic syndrome instead of diabetes, hypertension, dyslipidemia and waist circumference, and otherwise included the same variables as model 1. The association between CCA-IMT-max and normalized plasma fructose peak intensity values were assessed with the Spearman’s rank correlation test. In the analysis of the institutional cohort, we compared the changes in plasma fructose levels before and after the introduction of CPAP treatment by the Wilcoxon signed-rank test. Patients’ backgrounds were compared by Fisher’s exact test. Correlations between changes in plasma fructose levels and the following factors were analyzed by Spearman’s test: baseline plasma fructose levels, minimum SpO_2_ during sleep at diagnosis, and total time of SpO2 < 80% during sleep. A *P*-value of < 0.05 was considered as significant.

## Results

### The Nagahama study participants

The Nagahama study included 9850 participants^18^. Of these, we could assess both the Acti-ODI3% and GC–MS data in 6403 participants. After excluding 30 patients who received treatment for OSA syndrome (OSAS), data from 6373 participants were used for the analysis. Based on the severity, 2518 participants had normal, 3051 had mild and 804 had moderate-severe SDB (Fig. S1). We found no differences in the time after the last meal between the groups (Table 1).

**Table 1:** Background data on study participants from the Nagahama Study.

	Normal	Mild	Moderate-Severe	*P*-value
Number	2518	3051	804	
Male sex, n	369 (14.7)	1173 (38.4)	488 (60.7)	< 0.001
Age, years	52.00 [42.00, 64.00]	65.00 [54.00, 71.00]	69.00 [63.00, 74.00]	< 0.001
Acti-ODI3%, events/hour	3.26 [2.44, 4.12]	8.05 [6.27, 10.44]	20.62 [17.25, 26.56]	< 0.001
BMI, kg/m^2^	20.60 [19.10, 22.40]	22.40 [20.50, 24.40]	24.50 [22.40, 26.83]	< 0.001
Time after the last meal, hour	14.00 [11.12, 15.00]	14.00 [11.00, 15.00]	14.00 [10.00, 15.00]	0.219
Current smoker	241 (9.6)	280 (9.2)	73 (9.1)	0.854
Never smoker	1932 (76.7)	2048 (67.1)	422 (52.5)	< 0.001
Diabetes, n	56 (2.2)	240 (7.9)	127 (15.8)	< 0.001
Hypertension, n	511 (20.3)	1339 (43.9)	558 (69.4)	< 0.001
Dyslipidemia	896 (35.6)	1519 (49.8)	465 (57.8)	< 0.001
Gout, n	15 (0.6)	119 (3.9)	53 (6.6)	< 0.001
Systolic BP, mmHg *	118.00 [109.00, 128.00]	126.00 [116.00, 137.00]	133.00 [124.00, 143.00]	< 0.001
Diastolic BP, mmHg *	76.00 [69.00, 83.00]	81.00 [75.00, 88.00]	85.00 [79.00, 92.00]	< 0.001
Total protein, g/dl	7.20 [6.90, 7.50]	7.20 [7.00, 7.50]	7.30 [7.00, 7.60]	< 0.001
Albumin, g/dl	4.30 [4.20, 4.40]	4.30 [4.10, 4.40]	4.30 [4.10, 4.40]	< 0.001
HDL-Cholesterol, mg/dl	71.00 [60.00, 82.00]	63.00 [53.00, 75.00]	58.00 [49.00, 70.00]	< 0.001
LDL-Cholesterol, mg/dl	114.00 [95.00, 134.00]	118.00 [99.00, 136.00]	114.00 [99.00, 135.00]	< 0.001
Triglycerides**,** mg/dl	68.00 [50.00, 95.00]	85.00 [63.00, 118.00]	97.00 [69.00, 138.00]	< 0.001
Aspartate aminotransferase, U/l	20.00 [17.00, 24.00]	22.00 [19.00, 26.00]	23.00 [20.00, 28.25]	< 0.001
Alanine aminotransferase, U/l	15.00 [12.00, 20.00]	18.00 [14.00, 24.00]	20.00 [15.00, 29.00]	< 0.001
γ-glutamyl transpeptidase, U/l	17.00 [13.00, 24.00]	21.00 [15.00, 33.00]	27.00 [19.00, 45.00]	< 0.001
Alkaline phosphatase, U/l	189.00 [153.00, 233.00]	208.00 [172.00, 251.00]	217.00 [181.00, 258.25]	< 0.001

Fisher’s exact test was used for the analysis of categorical data, and Mann–Whitney’s U-test was used for continuous data.

Data are noted as number (%) or median [lower, higher interquartile range] * Blood pressure data was not available for two participants.

Acti-ODI3%: actigraph-adjusted oxygen desaturation index for ≥ 3% desaturation, *BMI* body mass index, *BP* blood pressure.

### Identification of fructose as the SDB-related plasma metabolites

**Figure 1:**
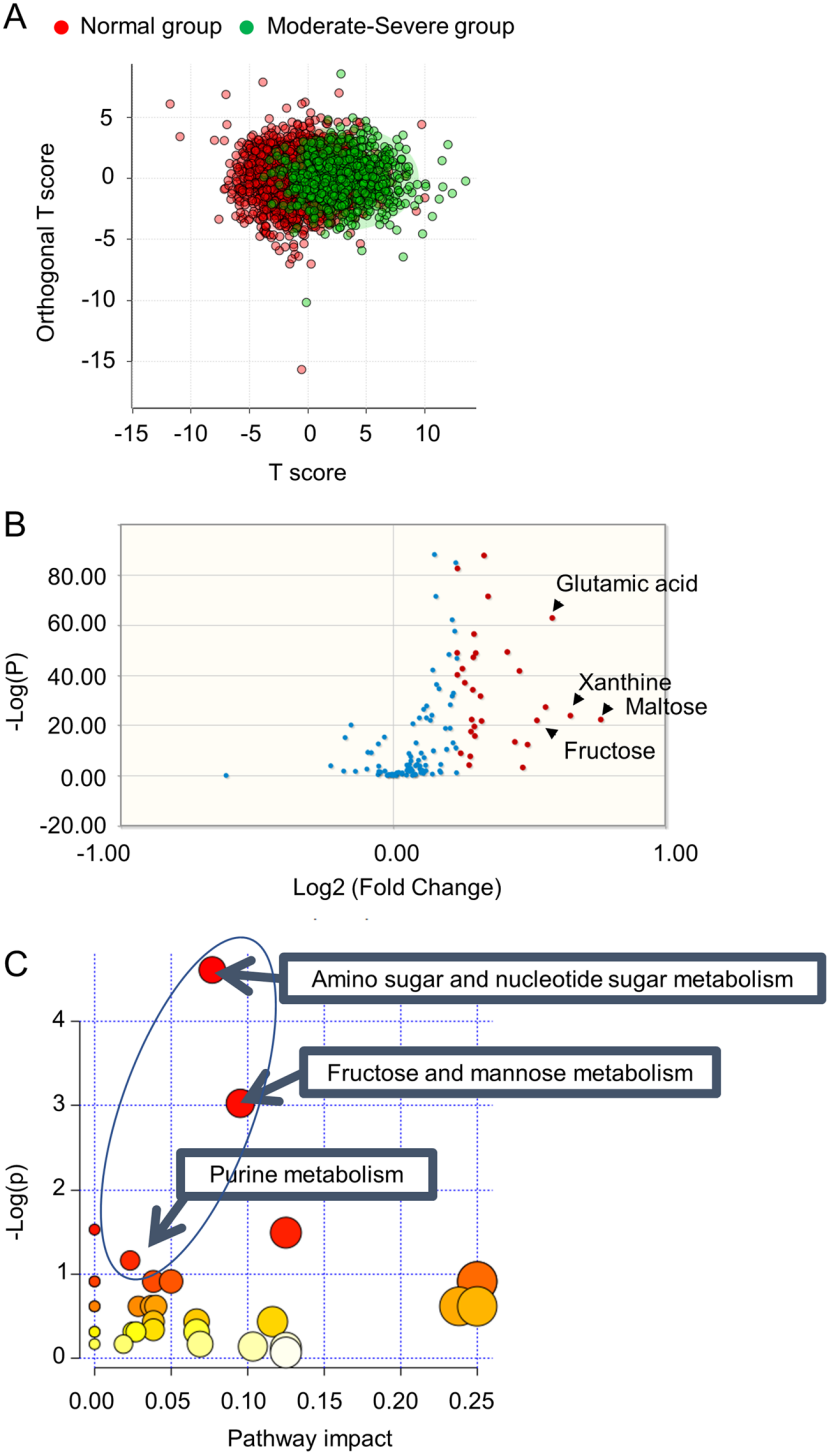
Identification of metabolic pathways associated with moderate-severe SDB. (**A**): Scatter plot based on T scores from the orthogonal partial least squares discriminant analysis. Red dots and green dots indicate participants in the normal-group and moderate-severe group, respectively. (**B**): Volcano plot based on fold-changes in normalized peak-intensity of each metabolite for the moderate-severe SDB group compared with the normal-group and its false discovery rate-adjusted *P*-value. Red dots indicate the metabolites with false discovery rate-adjusted *P*-values < 0.05 and ranked in the top 30 according to fold changes. (**C**): Pathways associated with the SDB-related metabolites shown in Table S3 in the online supplement. The circle indicates pathways which include fructose. *SDB* sleep disordered breathing.

First, we performed OPLS-DA by using the data from participants without SDB (n = 2518) and with moderate-severe SDB (n = 804). Data showed that the normal and moderate-severe groups were distributed in a partially overlapping manner in the T score-based scatter plot (Fig. 1A). A total of 38 metabolites showed a VIP score ≥ 0.9 (Table S1), and were considered as metabolites contributing to the differentiation of moderate-severe SDB patients from normal participants. We also compared the normalized metabolite peak intensity between the normal and moderate-severe SDB groups and extracted 30 metabolites as stated in the “Methods” section (Fig. 1B and Table S2). These two analyses identified 20 overlapping metabolites, and these metabolites were considered to be SDB-related (Table S3).

Next, pathway analysis was performed to examine the metabolic functions that were associated with SDB-related metabolites. Interestingly, we found that these metabolites were frequently related to the pathways associated with fructose (amino-sugar/nucleotide-sugar-metabolism, fructose/mannose-metabolism, and purine-metabolism) (Fig. 1C and Table S4). We further confirmed that plasma levels of fructose and its related metabolites, hypoxanthine and uric acid, elevated in accordance with the increased severity of SDB (Figs. S2 and S3). These results highlighted fructose as a characteristic metabolite in patients with SDB.

To evaluate if the association between SDB and plasma fructose levels was independent of participants’ background characteristics, we performed multivariate analyses with two models (Table 2). The analysis with Model 1 suggested that moderate-severe SDB was a significant factor influencing increased fructose levels (β: 0.210, standard error: 0.076, *P* = 0.006, generalized linear model). In contrast, neither diabetes, hypertension, dyslipidemia nor waist circumference was a significant factor. In model 2, we employed metabolic syndrome as an integrative variable that was substituted for diabetes, hypertension, dyslipidemia and waist circumference. In this analysis, moderate-severe SDB reproducibly showed a significant association with elevated plasma fructose levels (β = 0.196, standard error: 0.075, *P* = 0.009, generalized linear model), and metabolic syndrome was also a significant factor. Consistently, when participants with metabolic syndrome were stratified, we found that the plasma levels of fructose were significantly higher in moderate-severe SDB patients than in participants without SDB (Fig. S4). We further performed multivariate analyses for plasma levels of hypoxanthine and uric acid by using models with the same explanatory variables, and revealed that moderate-severe SDB was also significantly related to high hypoxanthine and uric acid levels. These results suggested that SDB was a specific factor for the increased plasma levels of fructose and its related metabolites.

**Table 2:** Associations between participants’ characteristics and plasma levels of fructose-associated metabolites.

Factor	Fructose			Hypoxanthine			Uric acid		
	β	Standard Error	*P*-value	β	Standard Error	*P*-value	β	Standard Error	*P*-value
Model 1									
Age	0.006	0.002	0.017	− 0.001	0.001	0.399	− 0.001	0.0003	0.010
BMI	0.006	0.011	0.177	0.018	0.004	< 0.001	0.010	0.002	< 0.001
Sex									
Pre-menopausal female	Reference			Reference			Reference		
Post-menopausal female	− 0.105	0.070	0.134	0.047	0.021	0.025	0.094	0.010	< 0.001
Male	− 0.034	0.071	0.637	0.203	0.021	< 0.001	0.312	0.010	< 0.001
Current smoker	− 0.038	0.072	0.600	0.015	0.022	0.487	0.002	0.010	0.859
Diabetes	0.087	0.083	0.293	− 0.019	0.025	0.433	− 0.013	0.012	0.268
Hypertension	0.035	0.048	0.466	0.027	0.014	0.062	0.033	0.006	< 0.001
Dyslipidemia	0.030	0.043	0.484	0.045	0.013	0.001	0.028	0.006	< 0.001
Waist circumference	0.001	0.004	0.736	− 0.001	0.001	0.023	0.002	0.0006	0.010
SDB severity									
Normal-group	Reference			Reference			Reference		
Mild-group	0.044	0.048	0.363	0.023	0.014	0.111	0.028	0.007	< 0.001
Moderate-severe group	0.210	0.076	0.006	0.050	0.023	0.030	0.027	0.011	0.021
Model 2									
Age	0.006	0.002	0.003	− 0.0002	0.001	0.725	− 0.0002	0.0003	0.487
BMI	0.006	0.007	0.361	0.016	0.002	< 0.001	0.015	0.001	< 0.001
Sex									
Pre-menopausal female									
Post-menopausal female	− 0.097	0.068	0.156	0.060	0.021	0.004	0.102	0.010	< 0.001
Male	− 0.042	0.070	0.548	0.208	0.021	< 0.001	0.316	0.010	< 0.001
Current smoker	− 0.041	0.071	0.566	0.017	0.022	0.432	0.003	0.010	0.773
Metabolic syndrome	0.271	0.089	0.002	0.019	0.027	0.477	0.046	0.013	< 0.001
SDB severity									
Normal-group									
Mild-group	0.047	0.047	0.322	0.023	0.014	0.109	0.029	0.007	< 0.001
Moderate-severe group	0.196	0.075	0.009	0.050	0.023	0.028	0.029	0.011	0.009

Analysis was performed by a generalized linear model by setting the link function as family = Gamma(log).

*BMI* body mass index, *SDB* sleep disordered breathing.

In addition, we found that the maximum value of intima-media thickness of the common carotid artery, an established surrogate marker of future CVD risk,^29,30^ was positively correlated with plasma fructose levels (R = 0.179, *P* < 0.001, Spearman’s rank correlation test). This significant correlation was observed for participants with and without SDB (Fig. S5).

### Impact of CPAP therapy on plasma fructose levels among patients with OSA

**Figure 2:**
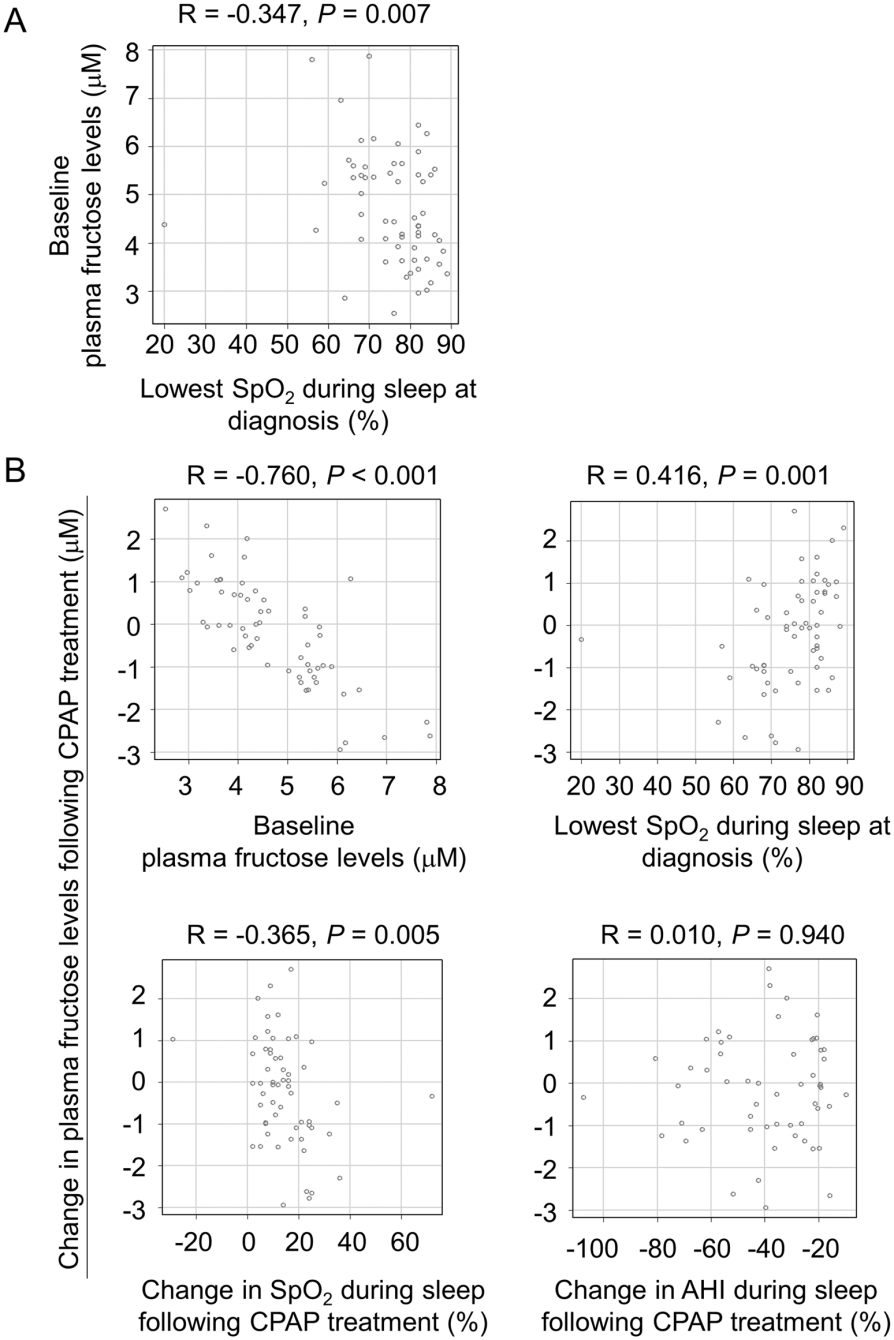
Associations between fructose levels and the degree of hypoxemia. (**A**): Association between baseline plasma fructose levels and lowest SpO_2_ levels during sleep at diagnosis. (**B**): Associations between changes in plasma fructose levels and baseline plasma fructose levels (upper left), lowest SpO_2_ levels during sleep at diagnosis (upper right), changes in lowest SpO_2_ levels during sleep (lower left) and changes in AHI (lower right). Each dot indicates one patient. Spearman’s rank correlation test was used.

Our institutional cohort included 60 patients. We analyzed the relationship between plasma fructose levels and several parameters during sleep. Before CPAP therapy, plasma fructose levels were significantly associated with the patients’ lowest SpO_2_ levels during sleep (R = − 0.347, *P* = 0.007, Fig. 2A, Spearman’s rank correlation test). Changes in fructose levels after three months of CPAP treatment showed a significant correlation with pre-treatment fructose levels (R = − 0.76, *P* < 0.001) and the lowest SpO_2_ levels during sleep (R = 0.416, *P* = 0.001) (Fig. 2B, upper panels, Spearman’s rank correlation test). In addition, we found that changes in SpO_2_ during sleep also showed a correlation, while changes in apnea–hypopnea index (AHI) did not show correlation (Fig. 2B, lower panels, Spearman’s rank correlation test). When classified based on the lowest median SpO_2_ levels, patients with lower SpO_2_ values had a significant decrease in plasma fructose levels after CPAP treatment, while those with higher SpO_2_ did not (Table 3).

**Table 3:** Comparison of institutional cohort obstructive sleep apnea patients with lower and higher lowest SpO_2_ levels during sleep.

	Lower SpO_2_	Higher SpO_2_	*P*-value
Number	33	27	
Age, years	57.00 [53.00, 66.00]	64.00 [60.00, 67.50]	0.021
Male sex, n (%)	29 (87.9)	22 (81.5)	0.718
BMI, kg/m^2^	27.90 [24.80, 33.20]	26.00 [24.60, 28.35]	0.095
Measurements of polysomnography at diagnosis			
Apnea hypopnea index, events/hour	51.50 [39.20, 66.80]	23.40 [21.10, 35.80]	< 0.001
Lowest SpO_2_ during sleep, min	70.00 [66.00, 76.00]	82.00 [82.00, 85.00]	< 0.001
Total time of SpO_2_ < 90% during sleep, min	18.40 [10.20, 51.80]	2.90 [0.80, 8.45]	< 0.001
Total time of SpO_2_ < 85% during sleep, min	10.90 [4.30, 32.30]	0.70 [0.10, 2.45]	< 0.001
Total time of SpO_2_ < 80% during sleep, min	1.20 [0.40, 5.90]	0.00 [0.00, 0.00]	< 0.001
Dyslipidemia, n (%)	21 (63.6)	19 (70.4)	0.784
Hypertension, n (%)	27 (81.8)	17 (63.0)	0.144
Diabetes, n (%)	7 (21.2)	4 (14.8)	0.739
Current smoker, n (%)	2 ( 6.1)	5 (18.5)	0.226
Plasma fructose levels, μM			
At diagnosis	5.27 [4.18, 5.64]	4.15 [3.51, 4.94]	0.012
At follow-up study	4.48 [3.96, 5.05]	4.49 [3.88, 4.93]	0.970
Changes between the diagnosis and follow-up*	− 0.49 [− 1.36, 0.30]	0.31 [− 0.52, 1.01]	0.013
Rate of days with ≥ 4 h/night CPAP usage (%)	67.0 [31.0, 88.0]	66.0 [23.95, 78.25]	0.431
Average usage of CPAP, h/night	267.0 [178.0, 350.0]	268.0 [154.0, 307.5]	0.494
Laboratory data at diagnosis			
Plasma glucose, mg/dl	97.00 [87.00, 104.00]	90.00 [84.00, 95.00]	0.019
Total protein, g/dl	6.90 [6.60, 7.40]	6.80 [6.50, 7.30]	0.383
Albumin, g/dl	4.00 [3.80, 4.10]	3.90 [3.65, 4.20]	0.828
HDL-Cholesterol, mg/dl	48.00 [43.00, 58.00]	51.00 [45.50, 57.50]	0.592
LDL-Cholesterol, mg/dl	116.00 [99.00, 133.00]	125.00 [103.00, 162.50]	0.188
Triglycerides, mg/dl	122.00 [88.00, 184.00]	138.00 [103.00, 185.00]	0.537
Aspartate aminotransferase, U/l	22.00 [17.00, 26.00]	22.00 [18.00, 28.50]	0.629
Alanine aminotransferase, U/l	21.00 [14.00, 28.00]	20.00 [16.50, 32.00]	0.598
γ-Glutamyl transpeptidase, U/l	30.00 [21.00, 52.00]	30.00 [26.00, 66.50]	0.298
Alkaline phosphatase, U/l	193.00 [173.00, 218.00]	195.00 [154.50, 221.00]	0.801
Lactate dehydrogenase, U/l	165.00 [150.00, 177.00]	160.00 [150.00, 178.50]	0.710
Uric acid, mg/dl	5.90 [5.00, 6.80]	6.20 [5.65, 6.55]	0.749

Fisher’s exact test was used for the analysis of categorical data, and Mann–Whitney’s U-test was used for continuous data. Data are noted as number (%) or median [lower, higher interquartile range].

*BMI* body mass index.

*P-values for changes in fructose levels before and after continuous positive airway pressure treatment were 0.319 for all patients, 0.028 for Lower SpO_2_ group and 0.202 for Higher SpO2 group. Wilcoxon signed-rank test was used for the analysis.

## Discussion

SDB is accompanied by a broad range of abnormalities in plasma metabolites. Among them, in this study, we have newly identified fructose as a critical metabolite related with SDB. SDB was significantly associated with elevated levels of plasma fructose, along with metabolic syndrome, even after the adjustment of participants’ background factors and complications. CPAP treatment was also found to lower plasma fructose levels, especially in OSA patients with high plasma fructose and low SpO_2_ levels during sleep.

Similar to our study, previous metabolomic studies of SDB patients revealed the alterations in a variety of plasma metabolites potentially related to hypoxia^8,14–16^, even though the contribution of concomitant metabolic disorders or patients’ background factors were not fully controlled due to their small number of study participants. In this study, taking advantage of a large-scale population-based study, we could successfully adjust for the confounding factors in SDB patients, and showed an independent association between moderate-severe SDB and plasma fructose levels. Importantly, complications such as diabetes, hypertension, dyslipidemia and visceral fat deposition represented by waist circumference were not significant factors for elevated plasma fructose levels. We consider that this result suggests the direct and unique relationship of SDB to elevated plasma fructose levels. On the other hand, we also found that metabolic syndrome, which meant the co-existence of multiple complications as shown above, was a significant factor for elevated plasma fructose levels. This result implies that the combination of these complications affects fructose metabolism differently from the effect of a single complication. As both SDB and metabolic syndrome were independently associated with plasma fructose levels, these conditions might act additively to the elevation of plasma fructose levels; however, further investigation is required to understand the detailed mechanism. We note that, during the review process of this paper, Zhang et al. reported associations between OSA and glutamate, oleoyl-linoleoyl-glycerol (18:1/18:2), linoleoyl-linoleoyl-glycerol (18:2/18:2), and phenylalanine by using large-scale cohorts^31^. We found a number of large differences in the factors of participants’ background between their dataset and our dataset, such as age, BMI, smoking status and racial background, which might have affected the results of the analyses. Future studies might examine metabolic features specific and universal among participants with different backgrounds.

Fructose, a species of sugar, has recently been focused as a metabolite with a strong association with CVD^32^. Fructose was shown to induce atherosclerosis by multiple mechanisms, and increased fructose consumption was correlated with higher rates of CVD^33,34^. Nevertheless, the conditions which are associated with elevated plasma fructose levels have not been reported with the exception of a study which suggested a high bood fructose levels in patients with diabetes mellitus^35^. Multivariate analyses in our study showed that the associations between SDB and plasma fructose levels were still significant after the adjustment for diabetes mellitus, indicating the associations that we found were independent from diabetes mellitus. Fructose is ingested as a natural ingredient in fruits and vegetables (< 9 g/day). However, people today consume substantially larger amounts of industrially manufactured fructose as sucrose or high-fructose corn syrup in various foods and beverages (> 70 g/day), and its impact on human health has attracted attention^32,36^. Fructose is rapidly converted into triglycerides in the liver^37^, causing dyslipidemia and visceral fat deposition, which lead to the increased risk of CVD. In addition, fructose actively glycates proteins and lipids, producing advanced glycation end products^38^, which directly increases the atherosclerosis risk^39^. Thus, fructose can accelerate the progression of atherosclerosis directly and indirectly^34^. This concept was proven by the recent epidemiological analyses that showed a positive association between fructose and cardiovascular risk^33^. In this study, we further revealed a positive correlation between plasm fructose levels and CCA-IMT-max, an established marker of increased risk of CVD. These data suggest that an increased fructose level is a potential underlying mechanism linking SDB and CVD^5,40^.

In this study, we also showed that OSA patients with severe hypoxemia during sleep had high levels of plasma fructose, which decreased after CPAP treatment. Changes in plasma fructose levels were significantly associated with changes in the lowest SpO_2_ levels, while the changes in AHI were not, suggesting that hypoxic conditions might contribute to the elevation of plasma fructose levels. Despite the adverse effects on CVD, fructose has an advantage as the energy source under conditions of limited oxygen availability^41^. Rodents residing in a hypoxic environment showed an upregulation of fructose metabolism^41^. Tissue hypoxia has been shown to activate fructose metabolism in mice and humans^42^. Therefore, it is tempting to hypothesize that intermittent hypoxia caused by SDB triggers the elevation of plasma fructose levels to increase its tissue availability^41^, which could get normalized following CPAP treatment. Indeed, recent data showed that the frequency and degree of hypoxemia were important in characterizing SDB^43^, possibly supporting our results. Thus, fructose levels in OSA patients could be normalized through treatment, although further investigation is needed.

This study suggests the potential significance of a therapeutic intervention targeting the SDB-fructose axis. For example, restricting the intake of fructose-containing foods (such as soft drinks) may be strongly recommended for patients with SDB, especially those with severe hypoxemia. Besides, inhibition of fructose uptake from the intestine may help prevent the progression of atherosclerosis progression in SDB patients. Future studies should address these questions and to establish the significance of fructose as a plasma biomarker for SDB.

This study has several limitations. First is that detailed information about food intake by participants in the Nagahama Study was not available. In order to minimize the effect of food intake on the data, the participants were instructed to skip the meal before their visit to the test site, and we found that there was no difference between the duration after the last meal, which were mostly longer than 10 h. However, nutritional intake by individual participants, such as the amount of daily calorie consumption, could not be adjusted for. On the other hand, in the institutional cohort, each patient was served meals in which components were carefully calculated according to meet the nutritional principles during the hospital stay. These meals remained the same before and after CPAP treatment, which suggests that nutrition intake was controlled in these patients. The second is that the institutional cohort included only patients with OSA; thus we could not evaluate the reproducibility of the results obtained in the Nagahama cohort, which requires the comparison between participants with and without SDB. Third, in the institutional cohort, the data required for the analysis of metabolic syndrome were lacking, which made it difficult to fully validate the data in the Nagahama cohort. To overcome these shortcomings, a future longitudinal study should be conducted that recruits participants both with and without SDB prospectively and collects comprehensive datasets and samples for the evaluation of metabolic status.

In conclusion, for the first time, we have identified a close relationship between fructose metabolism and SDB. Given the enormous impact of SDB on human health worldwide^4^, further investigations in combination with therapeutic interventions and analyses with novel parameters such as association analyses with multi-omics data are warranted to clarify the potential of fructose as a therapeutic target.

### Supplementary Information

Supplementary Information.

## Data Availability

The raw datasets in this study are available from the corresponding author on reasonable request.
