# Supplementary material for: Hyperfructosemia in sleep disordered breathing: metabolome analysis of Nagahama study
Source: Sci Rep. 2023 Aug 5;13:12735. doi: 10.1038/s41598-023-40002-1 (PMC10404271; doi:10.1038/s41598-023-40002-1)
Supplement: Supplementary file 1 — Supplementary Information. [file 41598_2023_40002_MOESM1_ESM.docx]

**Supplementary table S1. Variable of importance (VIP) scores for metabolites**

|  | VIP |
| --- | --- |
| Glucose | 1.7949 |
| Uric acid | 1.7948 |
| Isocitric acid | 1.7549 |
| Valine | 1.6448 |
| Indolelactic acid | 1.6207 |
| myo-Inositol | 1.5853 |
| Phenylalanine | 1.5344 |
| Glutamic acid | 1.5318 |
| Tyrosine | 1.4858 |
| 2-Hydroxyisovaleric acid | 1.3945 |
| Leucine | 1.3935 |
| Lactic acid | 1.3238 |
| Urea | 1.2557 |
| beta-Alanine | 1.2391 |
| 3-Hydroxyisobutyric acid | 1.2378 |
| Malic acid | 1.2354 |
| Fucose | 1.2319 |
| 2-Aminoadipic acid | 1.2318 |
| 3-Methyl-2-oxobutyric acid | 1.2003 |
| Mannose | 1.1967 |
| 3-Methyl-2-oxovaleric acid | 1.1946 |
| Ornithine | 1.1852 |
| Isoleucine | 1.1793 |
| Arabinose | 1.1575 |
| D-Threitol | 1.1501 |
| Alanine | 1.125 |
| 2-Oxoisocaproic acid | 1.1042 |
| Glucuronic acid | 1.0565 |
| Creatinine | 1.0542 |
| 3-Hydroxyisovaleric acid | 1.0502 |
| Lysine | 1.0164 |
| Maltose | 0.99609 |
| S-3,4-Dihydroxybutyric acid | 0.99088 |
| Arabitol | 0.98317 |
| Proline | 0.98161 |
| Fructose | 0.92748 |
| Xanthine | 0.90962 |
| Kynurenine | 0.88571 |
| Tryptophan | 0.88273 |
| 5-Oxoproline | 0.87173 |
| Aspartic acid | 0.85888 |
| Hypoxanthine | 0.84524 |
| Sucrose | 0.84272 |
| Erythritol | 0.83137 |
| Pyruvic acid | 0.81702 |
| Xylose | 0.80943 |
| Cystine | 0.78768 |
| 2-Oxoglutaric acid | 0.77869 |
| L-Methionine | 0.76826 |
| 2-Aminoethanol | 0.76298 |
| 2-Hydroxybutyric acid | 0.7464 |
| 3-Methylhistidine | 0.68203 |
| Indoleacetic acid | 0.68011 |
| 4-Hydroxyproline | 0.66783 |
| Ribulose | 0.62006 |
| Ribose | 0.5776 |
| Fumaric acid | 0.57585 |
| Gluconic acid | 0.56889 |
| Citrulline | 0.54968 |
| 2-Aminobutyric acid | 0.53977 |
| Glycolic acid | 0.51041 |
| scyllo-Inositol | 0.48383 |
| Glyceric acid 1,3-biphosphate | 0.46912 |
| Glycerol | 0.46738 |
| 2-Hydroxyisobutyric acid | 0.45923 |
| Phenol | 0.44699 |
| 2-Oxobutyric acid | 0.42069 |
| Xylitol | 0.3997 |
| D-2-Hydroxyglutaric acid | 0.38142 |
| Cysteine | 0.37064 |
| O-Phosphoethanolamine | 0.34897 |
| Uridine | 0.32766 |
| Allose | 0.32607 |
| Ribitol | 0.30857 |
| Indoxyl sulfate | 0.30694 |
| Hypotaurine | 0.2872 |
| Histidine | 0.24803 |
| 3-Aminoisobutyric acid | 0.23384 |
| Glutaric acid | 0.23243 |
| Glutamine | 0.23074 |
| Acetoacetic acid | 0.21471 |
| 3-3-Hydroxyphenyl-3-hydroxypropionic acid | 0.21055 |
| 2-Aminoethanesulfonic acid | 0.20372 |
| Myristic acid | 0.1932 |
| Elaidic acid | 0.19183 |
| Adipic acid | 0.18819 |
| Succinic acid | 0.17613 |
| Threonine | 0.16988 |
| Paraxanthine | 0.16599 |
| Lactitol | 0.16586 |
| Linoleic acid | 0.14923 |
| Pyrophosphate | 0.14922 |
| 1,6-Anhydro-beta-D-glucose | 0.14131 |
| Margaric acid | 0.12134 |
| Hippuric acid | 0.078863 |
| Oleic acid | 0.077199 |
| Cotinine | 0.076025 |
| Glyceric acid | 0.071411 |
| Oxalic acid | 0.057142 |
| Citric acid | 0.05456 |
| Palmitoleic acid | 0.051953 |
| Homocysteine | 0.047513 |
| Asparagine | 0.042136 |
| Aconitic acid | 0.014073 |
| Caproic acid | -9.06E-05 |
| 3-Indolepropionic acid | -0.01163 |
| 4-Cresol | -0.06478 |
| Boric acid | -0.10807 |
| 4-Hydroxybenzoic Acid | -0.13683 |
| 3-Hydroxybutyric acid | -0.18821 |
| Decanoic acid | -0.19281 |
| 1,5-Anhydro-D-sorbitol | -0.21146 |
| Caprylic acid | -0.21469 |
| Acetylglycine | -0.21785 |
| 1-Hexadecanol | -0.24057 |
| Threonic acid | -0.41955 |
| Benzoic acid | -0.43876 |
| Phosphoric acid | -0.54206 |
| Lauric acid | -0.5724 |
| Serine | -0.829 |
| Glycine | -0.96636 |

**Supplementary table S2. Top 30 metabolites ranked according to fold changes**

|  | Fold Change | log2(Fold change) | Adjusted *P*-value |
| --- | --- | --- | --- |
| Maltose | 1.6976 | 0.76352 | 6.01E-23 |
| Xanthine | 1.571 | 0.65168 | 1.69E-24 |
| Glutamic acid | 1.500 | 0.585 | 1.83E-63 |
| Glucuronic acid | 1.4748 | 0.56053 | 6.35E-28 |
| Fructose | 1.4426 | 0.52862 | 1.30E-22 |
| 3-Methylhistidine | 1.4086 | 0.4943 | 5.89E-13 |
| Allose | 1.3917 | 0.47681 | 0.00059291 |
| 2-Aminoadipic acid | 1.3798 | 0.46441 | 2.49E-42 |
| Xylose | 1.364 | 0.44783 | 4.90E-14 |
| 2-Hydroxyisovaleric acid | 1.3377 | 0.41979 | 6.77E-50 |
| Isocitric acid | 1.2738 | 0.34915 | 4.39E-72 |
| Uric acid | 1.2607 | 0.33424 | 2.21E-88 |
| S-3,4-Dihydroxybutyric acid | 1.2528 | 0.32521 | 2.29E-22 |
| D-Threitol | 1.2495 | 0.32131 | 2.82E-32 |
| Fucose | 1.2347 | 0.30413 | 1.79E-49 |
| Kynurenine | 1.2317 | 0.30065 | 2.23E-16 |
| Hypoxanthine | 1.2302 | 0.29887 | 3.64E-20 |
| Indolelactic acid | 1.2291 | 0.29757 | 4.77E-57 |
| beta-Alanine | 1.227 | 0.29517 | 9.05E-48 |
| Arabinose | 1.2254 | 0.29328 | 8.10E-35 |
| Arabitol | 1.2219 | 0.28918 | 6.01E-23 |
| Cystine | 1.2197 | 0.28655 | 3.74E-18 |
| Ribose | 1.2174 | 0.28377 | 2.13E-08 |
| Phenol | 1.214 | 0.27973 | 6.41E-05 |
| Isoleucine | 1.2007 | 0.26387 | 1.25E-37 |
| Creatinine | 1.1933 | 0.25497 | 3.10E-43 |
| Ribulose | 1.1881 | 0.24864 | 1.21E-09 |
| Tyrosine | 1.1785 | 0.2369 | 3.27E-83 |
| Lactic acid | 1.178 | 0.23632 | 8.82E-41 |
| Mannose | 1.1774 | 0.2356 | 1.48E-49 |

**Supplementary table S3. List of SDB-related metabolites**

| **Fold change in top 30 & FDR-P <0.05 & VIP >0.9** |
| --- |
| S-3,4-Dihydroxybutyric acid |
| 2-Aminoadipic acid |
| 2-Hydroxyisovaleric acid |
| Arabinose |
| Arabitol |
| beta-Alanine |
| Creatinine |
| D-Threitol |
| Fructose |
| Fucose |
| Glucuronic acid |
| Glutamic acid |
| Isocitric acid |
| Isoleucine |
| Lactic acid |
| Maltose |
| Mannose |
| Tyrosine |
| Uric acid |
| Xanthine |

**Supplementary table S4. Association of metabolic pathways and identified metabolites**

|  | Representative metabolite | Raw *P*-value | FDR-*P*-value | Impact |
| --- | --- | --- | --- | --- |
| Amino sugar and nucleotide sugar metabolism | D-Fructose | 0.009932 | 0.83427 | 0.07692 |
| Fructose and mannose metabolism | D-Fructose | 0.048418 | >0.99 | 0.09524 |
| Pentose and glucuronate interconversions | Glucuronic acid | 0.21686 | >0.99 | 0 |
| Ubiquinone and other terpenoid-quinone biosynthesis | Glutamic acid | 0.225 | >0.99 | 0.125 |
| Purine metabolism | Uric Acid | 0.31314 | >0.99 | 0.02326 |
| Ascorbate and aldarate metabolism | Glucuronic acid | 0.40158 | >0.99 | 0 |
| Inositol phosphate metabolism | Glucuronic acid | 0.40158 | >0.99 | 0 |
| Porphyrin and chlorophyll metabolism | Glutamic acid | 0.40158 | >0.99 | 0.03846 |
| Lysine degradation | 2-Aminoadipic acid | 0.40158 | >0.99 | 0.05 |
| Phenylalanine, tyrosine and tryptophan biosynthesis | Tyrosine | 0.40158 | >0.99 | 0.25 |
| Nitrogen metabolism | Glutamic acid | 0.40158 | >0.99 | 0.25 |
| Pyrimidine metabolism | beta-Alanine | 0.53968 | >0.99 | 0 |
| Phenylalanine metabolism | Tyrosine | 0.53968 | >0.99 | 0 |
| Glycolysis / Gluconeogenesis | Lactic Acid | 0.53968 | >0.99 | 0.02857 |
| Pyruvate metabolism | Lactic Acid | 0.53968 | >0.99 | 0.03704 |
| Starch and sucrose metabolism | D-Fructose | 0.53968 | >0.99 | 0.04 |
| beta-Alanine metabolism | beta-Alanine | 0.53968 | >0.99 | 0.2381 |
| D-Glutamine and D-glutamate metabolism | Glutamic acid | 0.53968 | >0.99 | 0.25 |
| Propanoate metabolism | beta-Alanine | 0.64729 | >0.99 | 0.03846 |
| Histidine metabolism | Glutamic acid | 0.64729 | >0.99 | 0.06667 |
| Tyrosine metabolism | Tyrosine | 0.64729 | >0.99 | 0.11628 |
| Glyoxylate and dicarboxylate metabolism | Isocitric acid | 0.71292 | >0.99 | 0.03846 |
| Galactose metabolism | D-Fructose | 0.73082 | >0.99 | 0 |
| Pantothenate and CoA biosynthesis | beta-Alanine | 0.73082 | >0.99 | 0 |
| Arginine and proline metabolism | Glutamic acid | 0.73082 | >0.99 | 0.025 |
| Glutathione metabolism | Glutamic acid | 0.73082 | >0.99 | 0.02703 |
| Butanoate metabolism | Glutamic acid | 0.73082 | >0.99 | 0.06667 |
| Valine, leucine and isoleucine biosynthesis | Isoleucine | 0.84519 | >0.99 | 0 |
| Valine, leucine and isoleucine degradation | Isoleucine | 0.84519 | >0.99 | 0.01887 |
| Citrate cycle (TCA cycle) | Isocitric acid | 0.84519 | >0.99 | 0.06897 |
| Aminoacyl-tRNA biosynthesis | Tyrosine | 0.86996 | >0.99 | 0.10344 |
| Arginine biosynthesis | Glutamic acid | 0.88336 | >0.99 | 0.125 |

**Supplementary figure S1. Flowchart of study participant selection**


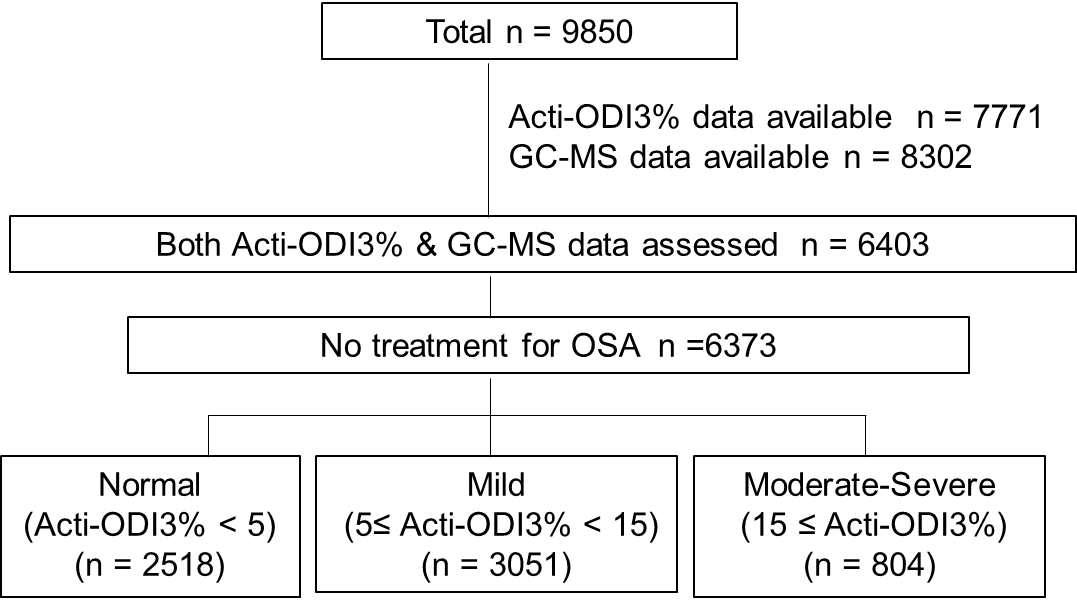


Acti-ODI3%: actigraph-adjusted oxygen desaturation index 3%, GC-MS: gas-chromatography mass-spectrometry, OSA: obstructive sleep apnea.

**Supplementary figure S2. Overview of fructose metabolism.**


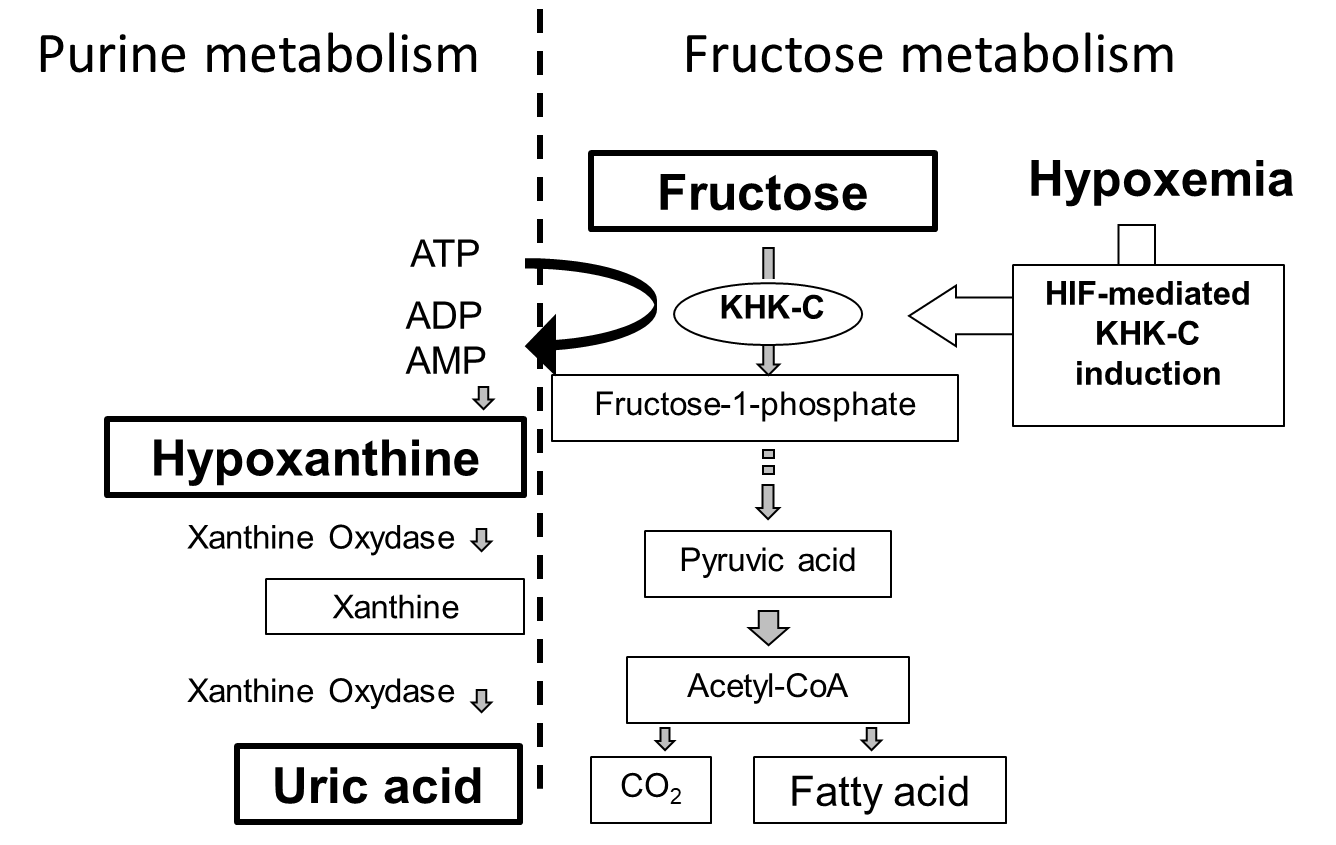


**Supplementary figure S3. Comparison of plasma levels of fructose and purine-associated metabolites among participants grouped according to the severity of SDB.**


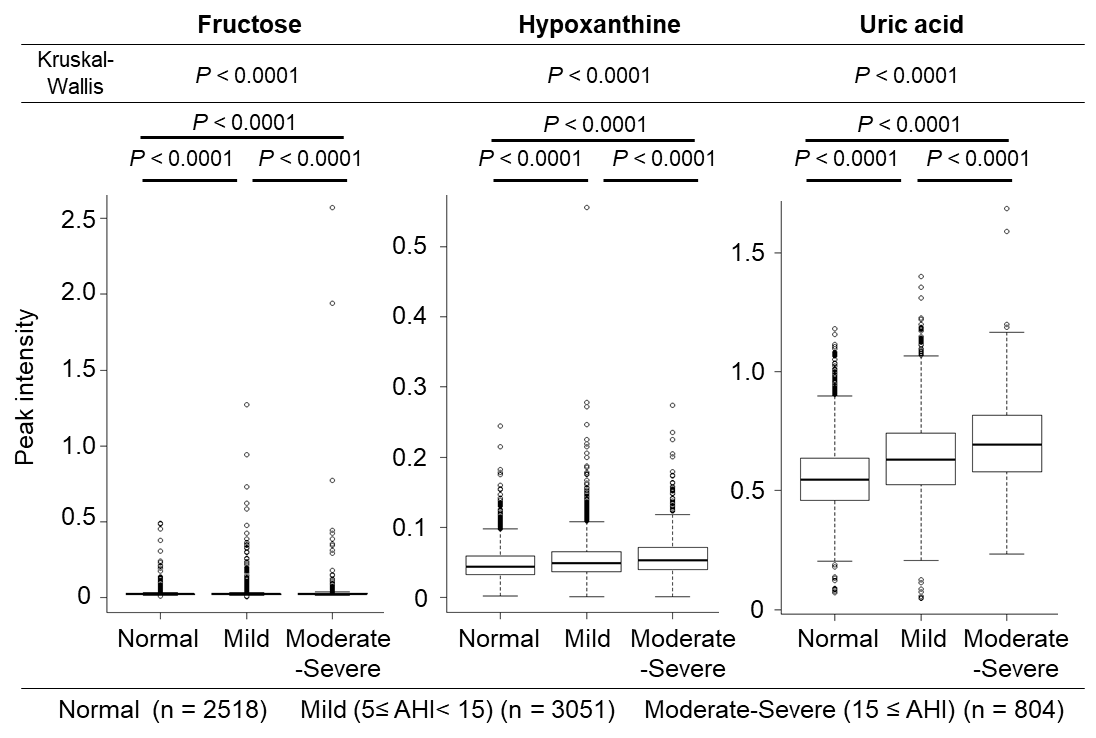


Comparisons of raw peak-intensity values for fructose (left), hypoxanthine (middle), and uric acid (right). Comparisons were performed between groups of participants according to the severity of SDB (normal, mild and moderate-severe groups).

Kruskal-Wallis test and Mann-Whitney U-test with adjustment for multiple comparisons by Holm’s method were used for the analysis.

**Supplementary figure S4. Comparison of plasma levels of fructose among participants with metabolic syndrome and grouped according to the severity of SDB.**


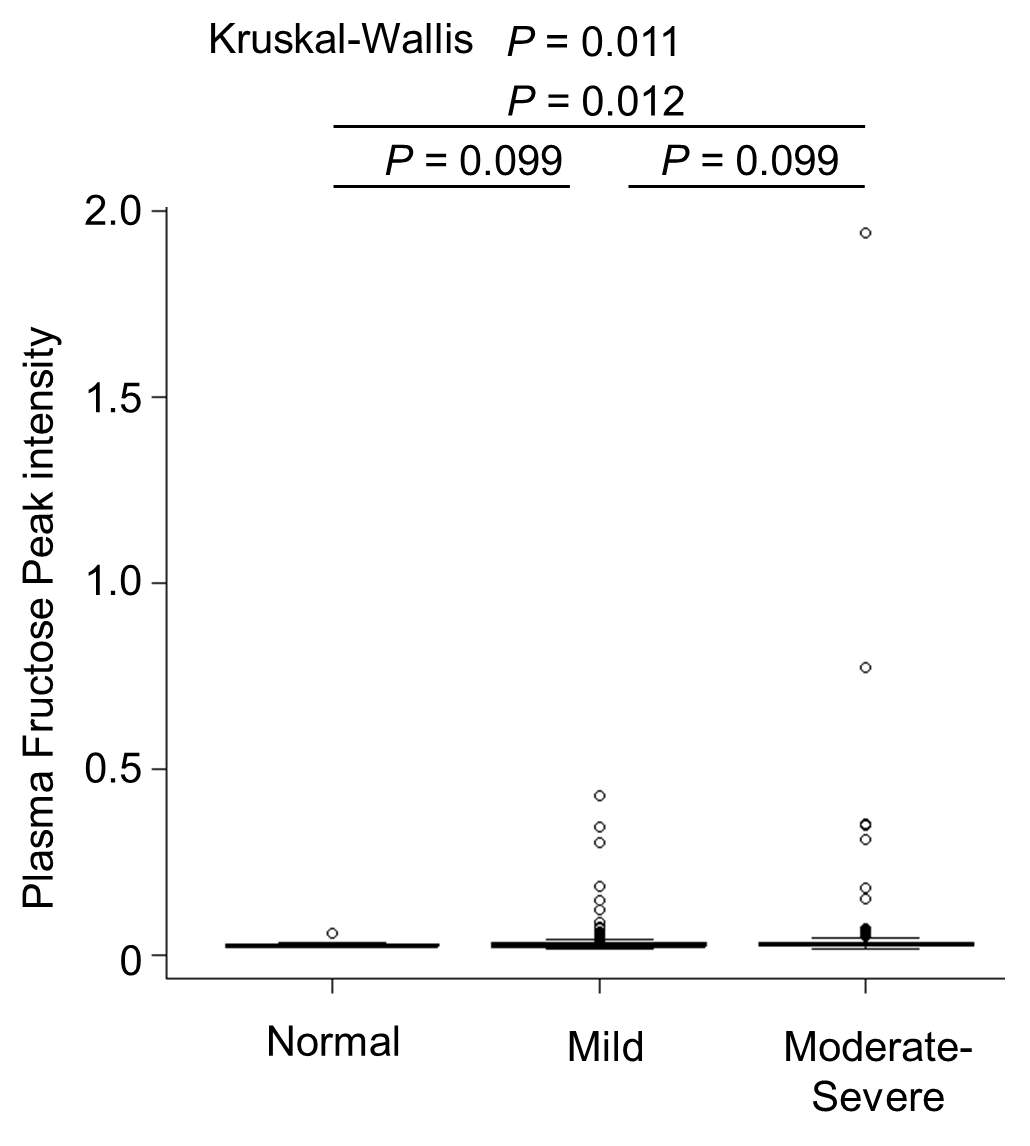


Comparisons were performed between groups of participants according to the severity of SDB (normal, mild and moderate-severe groups).

Kruskal-Wallis test and Mann-Whitney U-test with adjustment for multiple comparisons by Holm’s method were used for the analysis.

**Supplementary figure S5. Correlations between CCA-IMT-max values and plasma fructose levels**

**
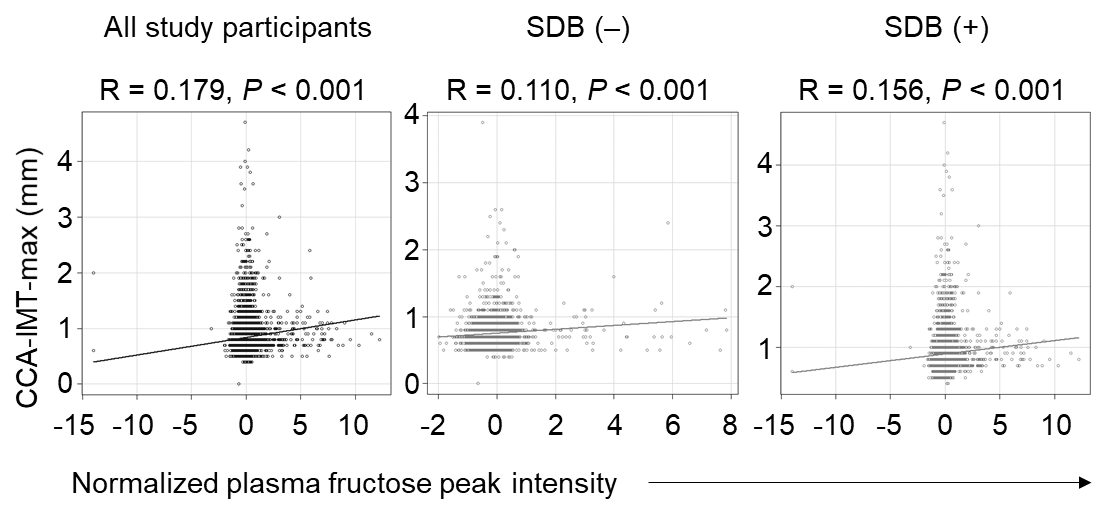
**

CCA-IMT-max values and raw peak intensity levels of plasma fructose were plotted. Each dot represents one participant. Compared with participants without SDB, participants with SDB tended to show higher fructose peak intensity levels and a higher correlation coefficient with CCA-IMT-max.

Spearman’s rank correlation test was used for statistical analysis.

SDB: sleep disordered breathing, CCA-IMT-max: the maximum value of intima-media thickness of the common carotid artery.
